# The Mechanism of *Andrographis paniculata* in the Treatment of Influenza Explored via Network Pharmacology and Molecular Docking

**DOI:** 10.1155/bmri/5752130

**Published:** 2025-12-21

**Authors:** Lan-ling Tian, Man-lin Zhang, Cong Wang

**Affiliations:** ^1^ Department of Microbiology and Immunology, School of Basic Medicine, Dali University, Dali, China, dali.edu.cn

**Keywords:** *Andrographis paniculata*, cytokines, influenza, molecular docking, network pharmacology

## Abstract

**Objective:**

The objective of this study is to investigate the potential mechanisms of *Andrographis paniculata* in treating influenza using network pharmacology and molecular docking approaches.

**Methods:**

The active components of *A. paniculata* were identified through the traditional Chinese medicine systems pharmacology database (TCMSP), and potential targets were predicted using SwissTargetPrediction. Gene targets associated with influenza were obtained from the GeneCards and OMIM databases. Venny 2.1.0 was used to create a Venn diagram to determine overlapping targets between *A. paniculata* and influenza. A “drug–component–target” interaction network was constructed using Cytoscape 3.7.2. A protein–protein interaction (PPI) network was developed with STRING 12.0 and visualized using Cytoscape 3.9.1 to identify core genes. Gene Ontology (GO) enrichment and Kyoto Encyclopedia of Genes and Genomes (KEGG) pathway analyses were conducted via the DAVID database, and the results were visualized using an online bioinformatics platform. Molecular docking was performed between major components and core targets using AutoDock 4.2.6 software.

**Results:**

A total of 24 active components of *A. paniculata* were identified, yielding 646 predicted drug targets, 1876 influenza‐associated gene targets, and 176 intersecting targets. GO enrichment analysis revealed 919 terms, primarily related to inflammatory responses and protein phosphorylation. KEGG analysis identified 173 enriched pathways, notably those related to lipid metabolism, atherosclerosis, and cancer. The principal active compounds demonstrated strong binding affinities with the core targets.

**Conclusion:**

*A. paniculata* may exert therapeutic effects against influenza by acting on core targets, such as TNF, IL‐6, AKT1, GAPDH, and STAT3. These findings provide a scientific foundation for the application of traditional Chinese medicine in the treatment of influenza.

## 1. Introduction

Influenza, also known as epidemic flu, is a major infectious disease characterized by clinical symptoms such as high fever, fatigue, headache, cough, and generalized muscle aches. It is an acute respiratory infection caused primarily by influenza A and B viruses [1, 2]. According to the World Health Organization, there are approximately 3–5 million severe cases globally each year, with an estimated 250,000–500,000 deaths resulting from influenza‐related respiratory illnesses. Vaccination remains the most effective strategy for controlling influenza. The incidence of influenza varies across regions, populations, and age groups, with children, the elderly, pregnant women, and other immunocompromised individuals being particularly susceptible. The genetic variability of influenza viruses can affect the early effectiveness of antiviral treatments. However, traditional Chinese medicine offers a holistic approach that targets multiple biological pathways, thereby exerting anti‐inflammatory, antipyretic, and antiviral effects [3–5].

*Andrographis paniculata* is the dried aerial part of the plant *A. paniculata* (family Acanthaceae), commonly referred to as the “King of Bitterness.” Native to Taiwan, China, and India, it is widely distributed in subtropical regions, including India, Thailand, Vietnam, and China. It is bitter in taste and cold in nature, and it is associated with the heart, lung, large intestine, and bladder meridians. Its pharmacological properties include antibacterial, antitumor, antiviral, cardioprotective, hypoglycemic, antiplatelet, and hepatoprotective activities. It has been traditionally used to treat conditions such as upper gastrointestinal and respiratory tract infections, fever, herpes, and diabetes [6–9]. In traditional Vietnamese medicine, it is employed for the treatment of influenza, cough, pneumonia, tonsillitis, urethritis, and inflammatory bowel disease. The leaves primarily contain diterpene lactones, whereas the roots are rich in flavonoids [10]. Extracts of *A. paniculata* have demonstrated broad‐spectrum antiviral activity and efficacy against inflammation caused by herpes simplex virus Type 1 and the influenza virus [11].

Influenza remains a significant global health concern, and traditional herbal medicines such as *A. paniculata* have shown potential therapeutic benefits. Therefore, in this study, network pharmacology and molecular docking methods were used to predict the active components of *A. paniculata* and its core targets in the treatment of influenza. The objective was to explore the underlying mechanisms, thereby establishing a theoretical foundation and providing experimental evidence for the practical application and further development of *A. paniculata* in influenza therapy.

## 2. Materials and Methods

### 2.1. Screening Active Components/Target Sites of *A. paniculata* and Identifying Influenza‐Related Genetic Targets

Taking oral bioavailability (OB) ≥ 30% and drug similarity (DL) ≥ 0.18 as the screening criteria, the effective components of *A. paniculata* were identified by using the database of systematic pharmacology of traditional Chinese medicine (TCMSP) [12], while using SMILES structure in the potential targets that were predicted through SwissTargetPrediction (http://www.swisstargetprediction.ch/) [13]. Gene targets associated with influenza were retrieved from the GeneCards (https://www.genecards.org/) [14] and OMIM databases (https://mirror.omim.org) [15]. Duplicated entries were eliminated to generate a comprehensive set of disease‐related gene targets [16].

### 2.2. Construction of the *A. paniculata*–Component–Target Network

The screened active components and their corresponding targets were used to construct a “drug–component–target” interaction network in Cytoscape 3.7.2.

### 2.3. Identification of Overlapping Targets Between *A. paniculata* and Influenza

Venny 2.1.0 was employed to generate a Venn diagram illustrating overlapping targets. Intersection targets between *A. paniculata* and influenza were identified using Venny 2.1.0 (https://bioinfogp.cnb.csic.es/tools/venny/), generating a Venn diagram to illustrate shared targets.

### 2.4. Construction of the Protein–Protein Interaction Network

In order to determine the core target, a drug‐component–target network and a protein–protein interaction (PPI) network were constructed using Cytoscape 3.9.1, a software capable of calculating various parameters of individual nodes in a network graph. Key hub genes were identified with PPI data supported by STRING 12.0, a database (https://cn.string-db.org/) that contains most known human PPI information. We selected multiple proteins and imported the intersection targets of *Coptis chinensis* and influenza into a Venn diagram. The organism field was set to “Homo sapiens.” The minimum required interaction confidence was set to 0.400, unconnected nodes were hidden, and other parameters remained unchanged by default. The resulting TSV file was exported and imported into Cytoscape 3.9.1. Network metrics including “Betweenness,” “Closeness,” and “Degree” were applied for target filtering. Final core targets were identified based on values exceeding the thresholds for all three metrics, and the PPI network diagram was constructed accordingly [17, 18].

### 2.5. GO and KEGG Bioenrichment Analysis

Gene Ontology (GO) and Kyoto Encyclopedia of Genes and Genomes (KEGG) pathway enrichment analyses were performed using the DAVID database (https://david.ncifcrf.gov/). After selecting “Start Analysis” the intersection gene set of *A. paniculata* and influenza was imported [19]. The “Identifier” was set to “OFFICIAL_GENE_SYMBOL,” the “Gene List” box was checked, and “Homo sapiens” was selected as the species. After completing the database search, the GOTERM_BP, GOTERM_CC, GOTERM_MF, and KEGG_PATHWAY results were downloaded. The Top 20 entries based on enrichment percentage were selected and visualized using Weisenx in the form of bubble charts. These charts were used to identify the primary functions of the target proteins and the related signaling pathways.

### 2.6. Molecular Docking

The active ingredients were retrieved from the TCMSP database using their PubChem CID or molecule name. These identifiers were used to locate and download the 2D structure files in SDF format from the PubChem database (https://pubchem.ncbi.nlm.nih.gov/). The SDF files were then imported into ChemBio3D software to optimize and convert the molecules into 3D structures, and exported in mol2 format. The core protein targets were identified using the UniProt database and selected based on established literature criteria. The corresponding 3D structures of the macromolecular receptors were downloaded from the Protein Data Bank (PDB; https://www.rcsb.org/) in PDB format [20]. These PDB files were processed using PyMOL 3.1.0 software to remove water molecules and residues, and saved for further analysis. Ligand and receptor files were then converted to pdbqt format using AutoDock 4.2.6 tools. AutoDock 4.2.6 software was employed to define grid coordinates and perform molecular docking [21]. Visualization and analysis of the docking results were conducted using R language and PyMOL 3.1.0.

## 3. Result

### 3.1. Screening of Active Components/Targets in *A. paniculata* and Analysis of Influenza‐Related Gene Targets

A total of 24 active components of *A. paniculata* were identified, corresponding to 646 predicted drug targets. The active ingredients were shown in Table 1. Targets retrieved from the GeneCards human gene database were screened twice using a relevance score threshold greater than or equal to the median value. These were then combined with disease targets obtained from the OMIM database and de‐duplicated. A total of 1876 unique influenza‐related gene targets were identified.

**Table 1:** Main active Ingredients of *Andrographis paniculata.*

**Mol ID**	**Molecule Name**	**Oral bioavailability/%**	**Drug likeness**
MOL000173	Wogonin	30.68	0.23
MOL002928	Oroxylin a	41.37	0.23
MOL002932	Panicolin	76.26	0.29
MOL008203	14‐deoxy‐11‐oxo‐andrographolide	57.06	0.34
MOL008204	Mono‐O‐methylwightin	103.11	0.4
MOL008206	Moslosooflavone	44.09	0.25
MOL008209	Deoxycamptothecine	50.01	0.77
MOL008210	Deoxyelephantopin	105.32	0.40
MOL008213	14‐deoxy‐12‐methoxyandrographolide	70.29	0.36
MOL008215	Paniculide B	52.27	0.21
MOL008216	Paniculide C	79.73	0.21
MOL008217	Paniculogenin	47.66	0.75
MOL008218	1‐Monoolein	34.13	0.3
MOL008219	3‐[2‐[(1R,4aS,5R,8aS)‐5,8a‐dimethyl‐2‐methylene‐5‐methylol‐decalin‐1‐yl]ethyl]‐5H‐furan‐2‐one	51.78	0.28
MOL008222	Andrographidine B_qt	72.72	0.33
MOL008223	Andrographidine C	56.85	0.77
MOL008226	14‐deoxyandrographolide	56.3	0.31
MOL008228	Andrographin	37.57	0.33
MOL008229	Andrographin F	33.34	0.85
MOL008230	Andrographidine F_qt	77.13	0.45
MOL008232	(3Z,4S)‐3‐[2‐[(1R,4aS,5R,6R,8aS)‐6‐hydroxy‐5,8a‐dimethyl‐2‐methylene‐5‐methylol‐decalin‐1‐yl]ethylidene]‐4‐hydroxy‐tetrahydrofuran‐2‐one	46.96	0.36
MOL008234	Andrographolide‐19‐β‐D‐glucoside_qt	53.44	0.35
MOL008238	3‐[2‐[(1S,4aR,5S,8aR)‐5,8a‐dimethyl‐2‐methylene‐5‐methylol‐decalin‐1‐yl]ethyl]‐5H‐furan‐2‐one	63.54	0.28
MOL008239	Quercetin tetramethyl(3 ^′^,4 ^′^,5,7) ether	31.57	0.41

### 3.2. *A. paniculata*–Component–Target Network Diagram

**Figure 1:**
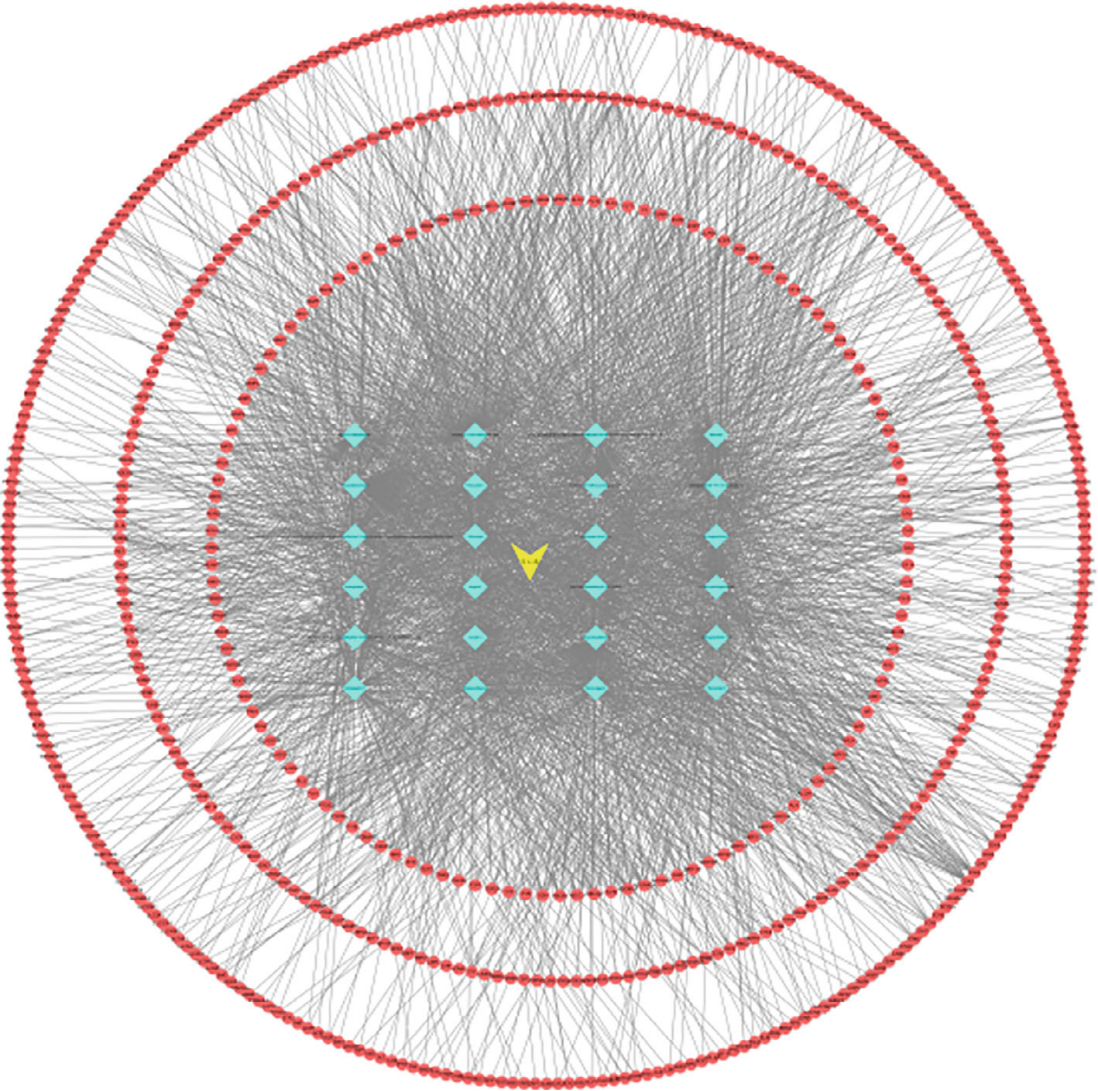
Network diagram of traditional Chinese medicine–components–targets.

Following data integration of the traditional Chinese medicine components and their targets, two files, “network.xlsx” and “type.xlsx,” were created and imported into Cytoscape 3.7.2 to construct the network diagram. The resulting graph contained 175 nodes and 6502 edges, as shown in Figure 1. In the network, red nodes represented target genes, blue nodes represented the 24 active components, and yellow nodes indicated the traditional Chinese medicine name. This diagram highlighted the multitarget and multipathway pharmacological characteristics of *A. paniculata*.

### 3.3. Construction of PPI Network and Core Target Screening Analysis

**Figure 2:**
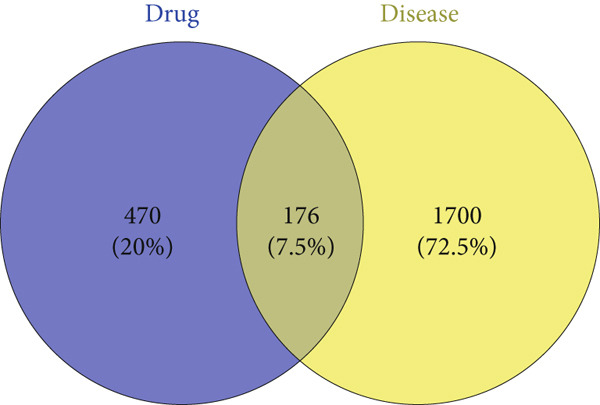
Drug–disease target Venn diagram.

**Figure 3:**
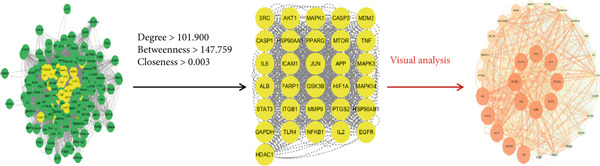
Protein–protein interaction network diagram.

The intersection of *A. paniculata* and influenza target genes was identified using Venny 2.1.0, yielding 176 common targets. A Venn diagram was generated, as shown in Figure 2, to visualize the overlap. These overlapping genes represent the potential therapeutic targets of *A. paniculata* against influenza. The intersection targets were then imported into the STRING 12.0 database for PPI analysis. The resulting interaction data were exported in TSV format and loaded into Cytoscape 3.9.1. The CentiScaPe 2.2 plug‐in was used to calculate the “Betweenness,” “Closeness,” and “Degree” centrality metrics [22]. Nodes were filtered sequentially based on values exceeding the median for each metric. This process yielded a refined network with 31 nodes and 876 edges. A visualization of the PPI network was shown in Figure 3, which included the core target screening diagram, the core target selection diagram, and the final network drawing. The five nodes with the highest degree values, namely, TNF, IL6, AKT1, GAPDH, and STAT3, were identified as core targets. In the visualizations, nodes with higher degree values were represented by larger circles, thicker connecting edges, and brighter colors, indicating their greater likelihood of serving as key targets in the treatment of influenza.

### 3.4. GO and KEGG Bioenrichment Analysis

The 176 overlapping gene targets were submitted to the DAVID database for functional enrichment analysis. Based on *p* value rankings, the results were categorized into three GO functional domains: biological process (BP), cellular component (CC), and molecular function (MF). The top 20 enriched terms for each category were selected and visualized as bubble plots using the Weishengxin online data platform. The results were shown in Figures 4a, 4b, and 4c. In the plots, the size of each bubble reflected the number of genes involved, whereas the bubble color intensity corresponded to the *p* value (with redder colors indicating smaller *p* values and higher significance). The BPs were primarily related to inflammatory responses and protein phosphorylation. CC analysis indicated significant enrichment in cytosol, cytoplasm, nucleus, plasma membrane, and related substructures. Key MFs included protein binding, ATP binding, protein kinase activity, threonine kinase activity, and serine/threonine kinase activity.

Figure 4Bubble charts representing the GO and KEGG pathway enrichment analysis of *Andrographis paniculata* in the treatment of influenza: (a) biological process (BP); (b) cellular component (CC); (c) molecular function (MF); (d) KEGG pathway enrichment.

**Figure:**
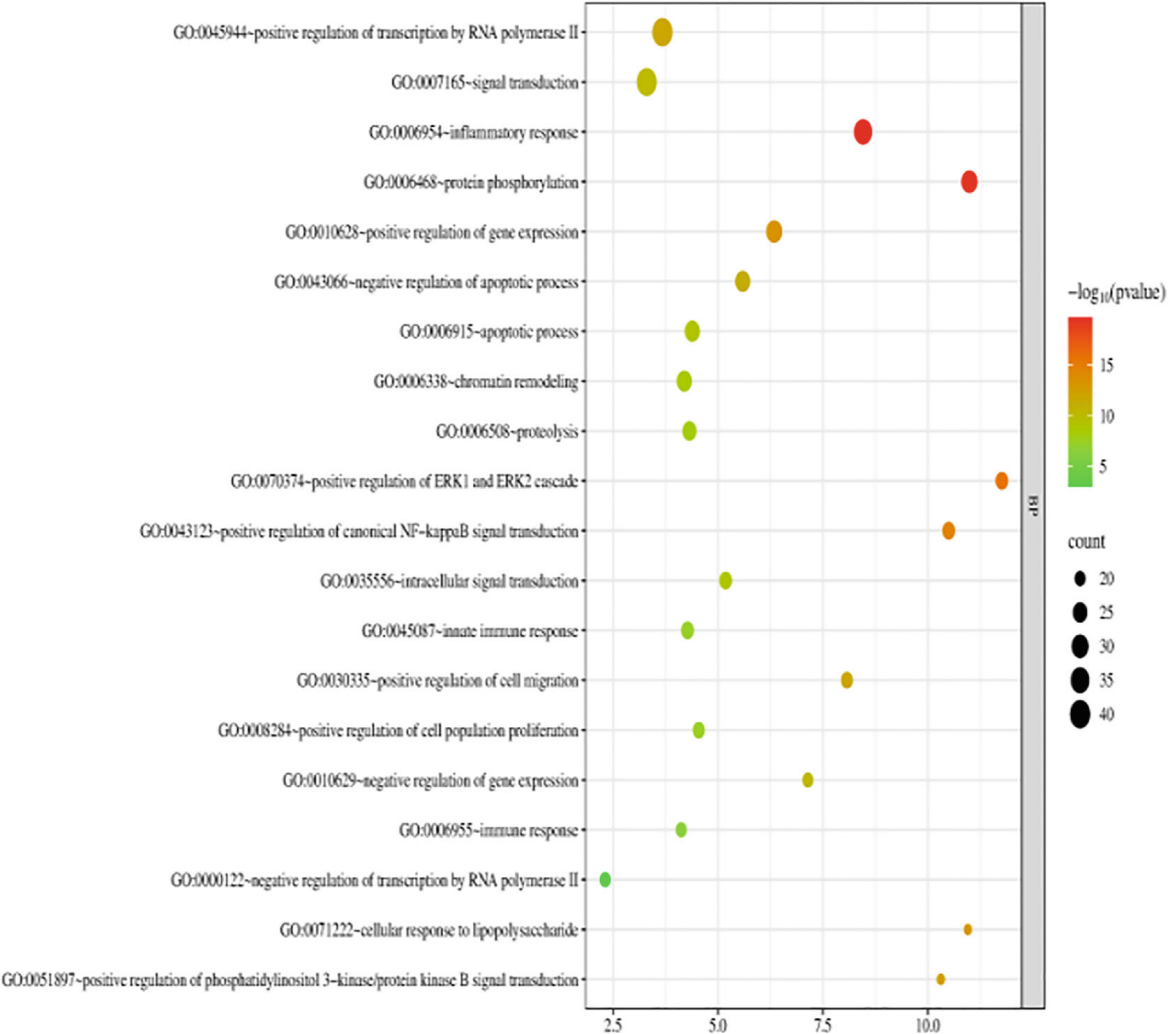
(a)

**Figure:**
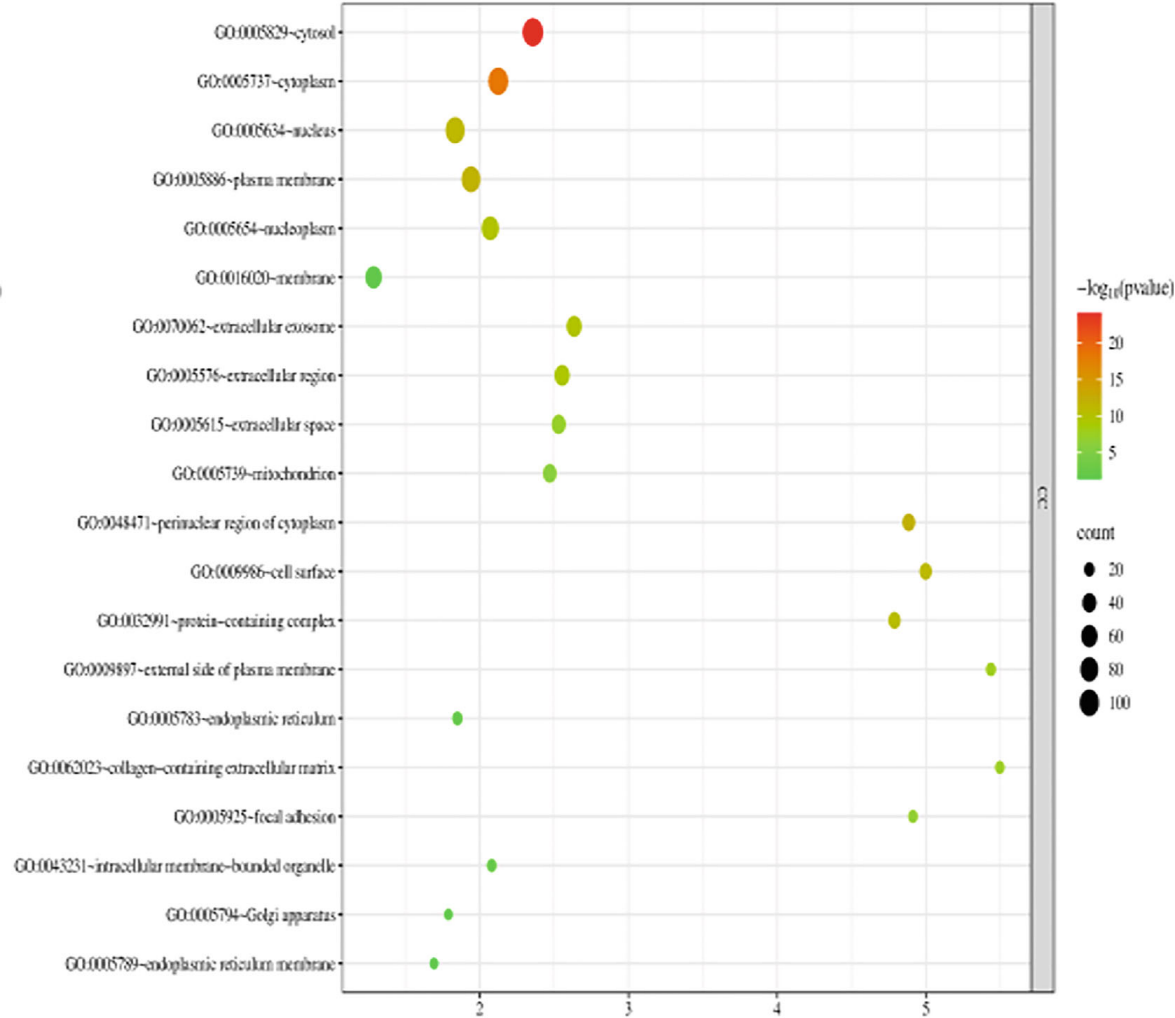
(b)

**Figure:**
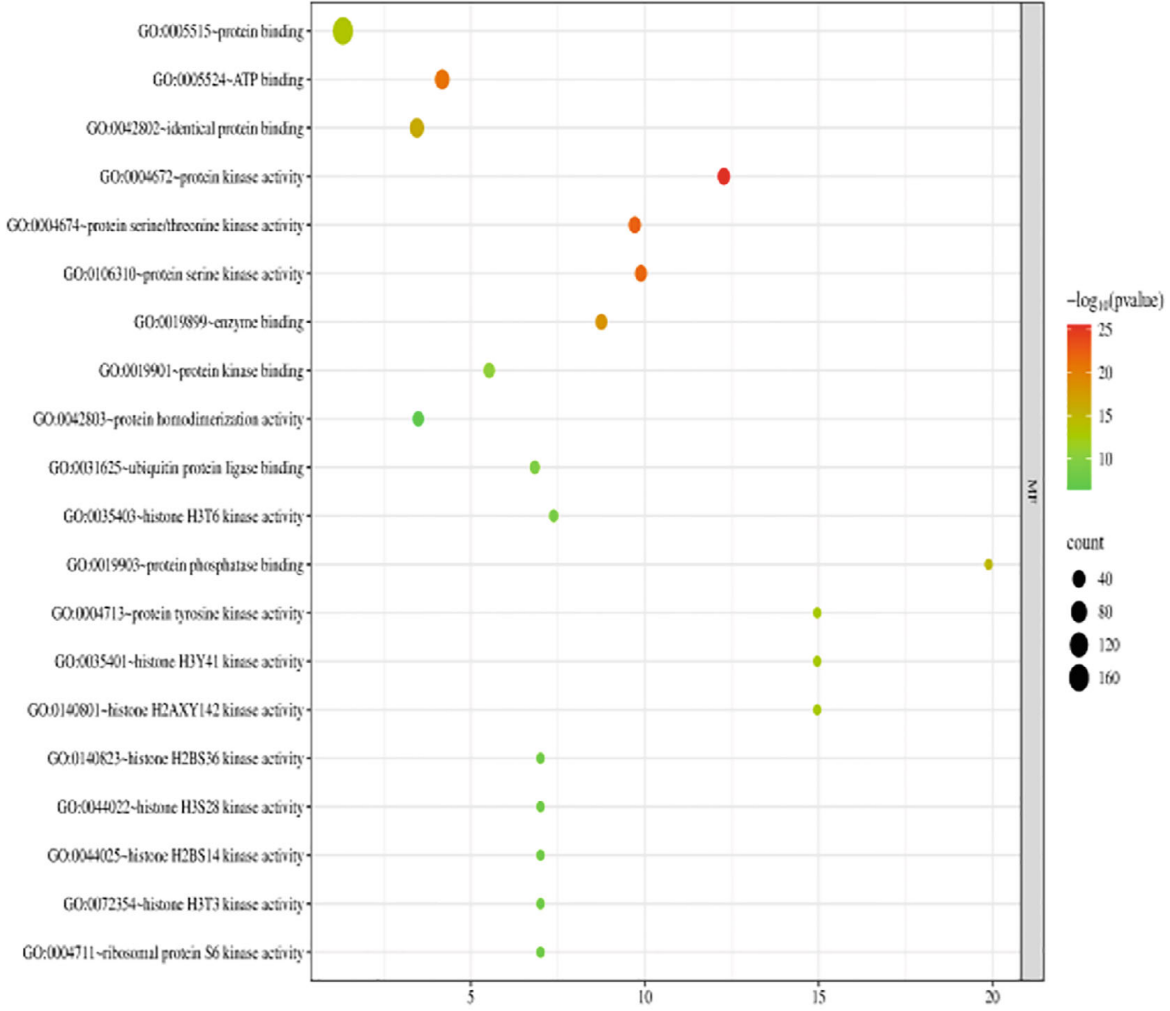
(c)

**Figure:**
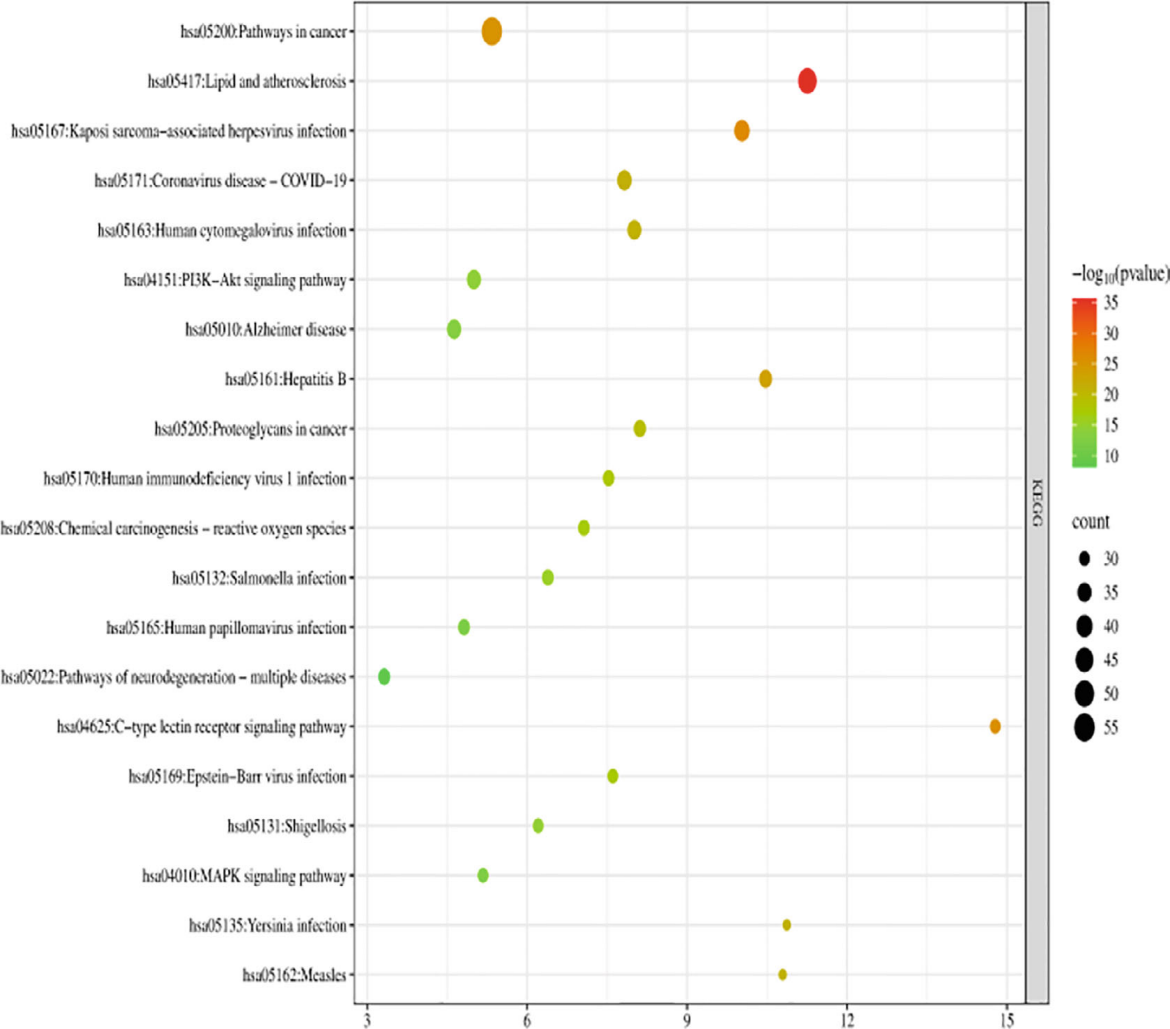
(d)

KEGG pathway analysis identified 173 enriched pathways, particularly those involved in lipid metabolism, atherosclerosis, and cancer‐related signaling. The Top 20 pathways, ranked in ascending order of *p* values, were presented in the form of a bubble chart in Figure 4d. The size of the bubbles represented the number of enriched genes, and the color reflected the negative logarithm of the *p* value (−log10 [*p* value]). The redder the color, the more significant the statistical enrichment.

### 3.5. Analysis of Molecular Docking Results

The five core targets, namely, TNF, IL6, AKT1, GAPDH, and STAT3, identified based on the highest degree values in the PPI network were selected for molecular docking validation. These targets were docked with the top four active components of *A. paniculata*, which were ranked by their own degree values. The results of molecular docking showed that all of the binding energies were negative, ranging from −4.4 to −8.0 kcal/mol, indicating stable binding affinities between the active compounds and core targets. The detailed binding energy values were presented in Table 2. For each core target, the two compound‐target pairs with the strongest binding affinities were selected and visualized using PyMOL 3.1.0 software. The molecular docking visualizations were shown in Figures 5a, 5b, 5c, 5d, and 5e

**Table 2:** Binding energies between core targets and active component molecules.

**Core target**	**Active ingredient**	**Binding energy(kcal/mol)**
TNF	Andrographin F	−7.7
TNF	Andrographidine C	−7.5
TNF	Andrographin	−5.5
TNF	Mono‐O‐methylwightin	−6.7
IL6	Andrographin F	−5.6
IL6	Andrographidine C	−5.6
IL6	Andrographin	−5.4
IL6	Mono‐O‐methylwightin	−5.1
AKT1	Andrographin F	−4.5
AKT1	Andrographidine C	−4.5
AKT1	Andrographin	−4.4
AKT1	Mono‐O‐methylwightin	−4.5
GAPDH	Andrographin F	−5.0
GAPDH	Andrographidine C	−7.4
GAPDH	Andrographin	−7.8
GAPDH	Mono‐O‐methylwightin	−8.0
STAT3	Andrographin F	−5.7
STAT3	Andrographidine C	−6.7
STAT3	Andrographin	−6.3
STAT3	Mono‐O‐methylwightin	−6.2

Figure 5Molecular docking visualization results: (a) TNF with Andrographin F and Andrographidine C; (b) IL6 with Andrographin F and Andrographidine C; (c) AKT1 with Andrographin F and Mono‐O‐methylwightin; (d) GAPDH with Mono‐O‐methylwightin and Andrographin; (e) STAT3 with Andrographidine C and Andrographin.

**Figure:**
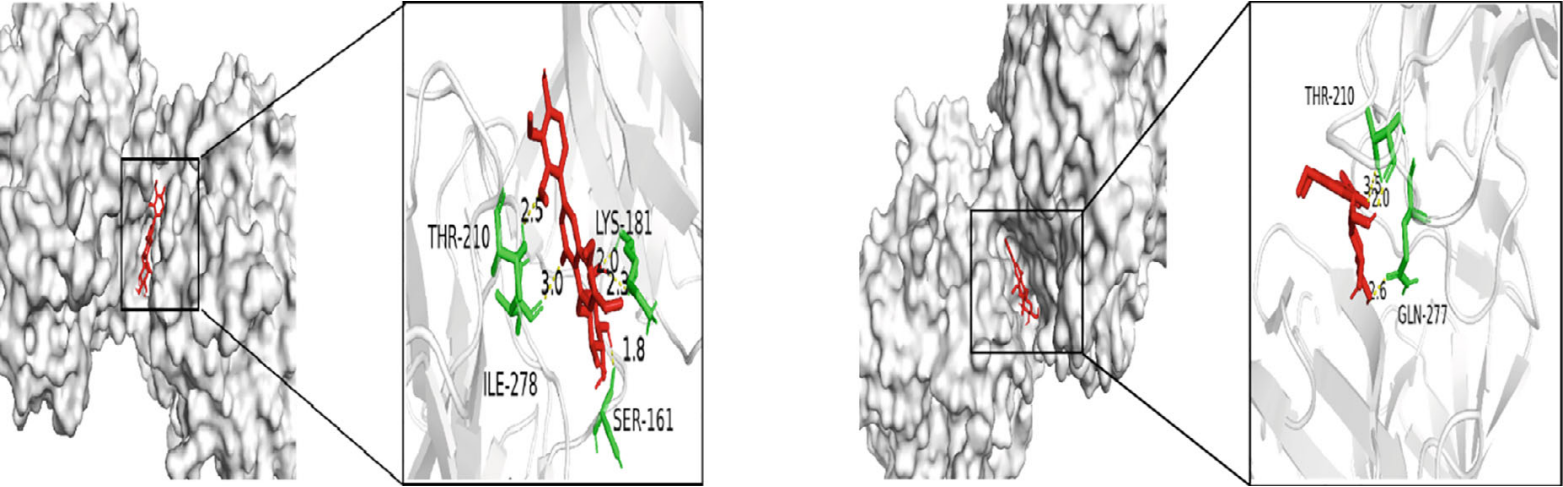
(a)

**Figure:**
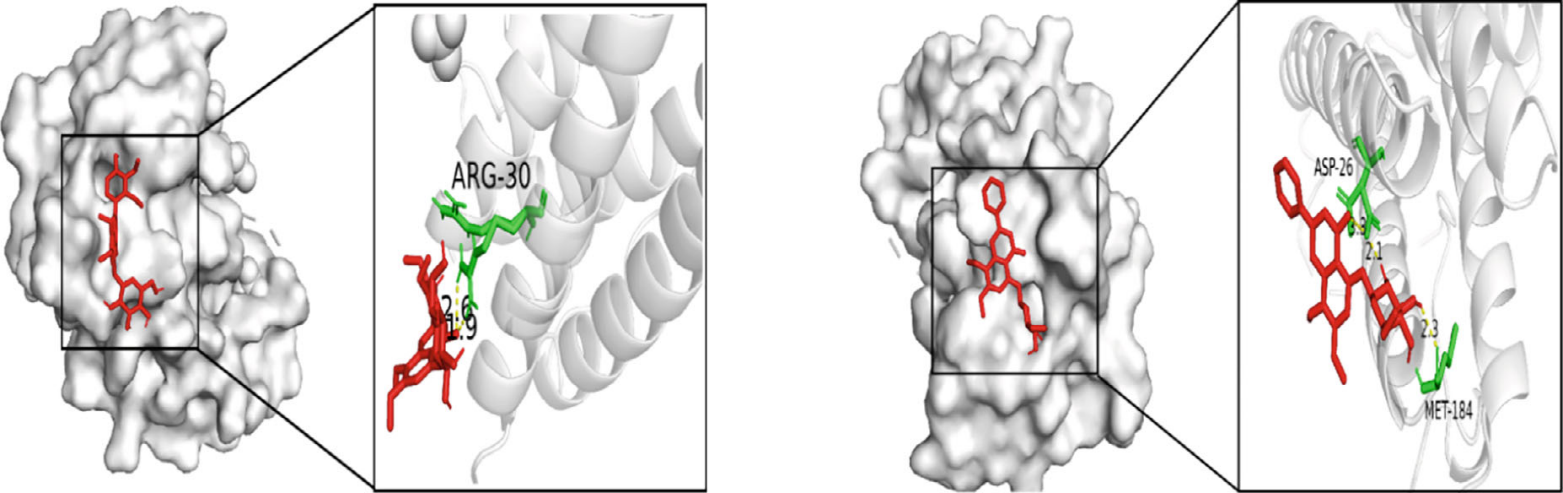
(b)

**Figure:**
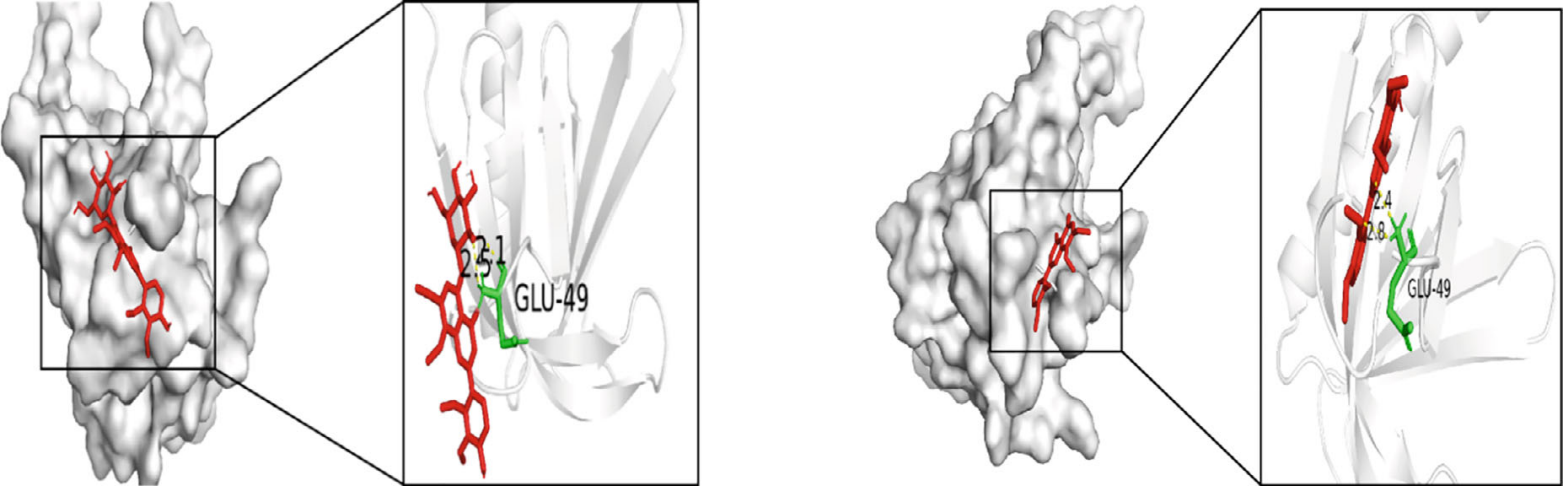
(c)

**Figure:**
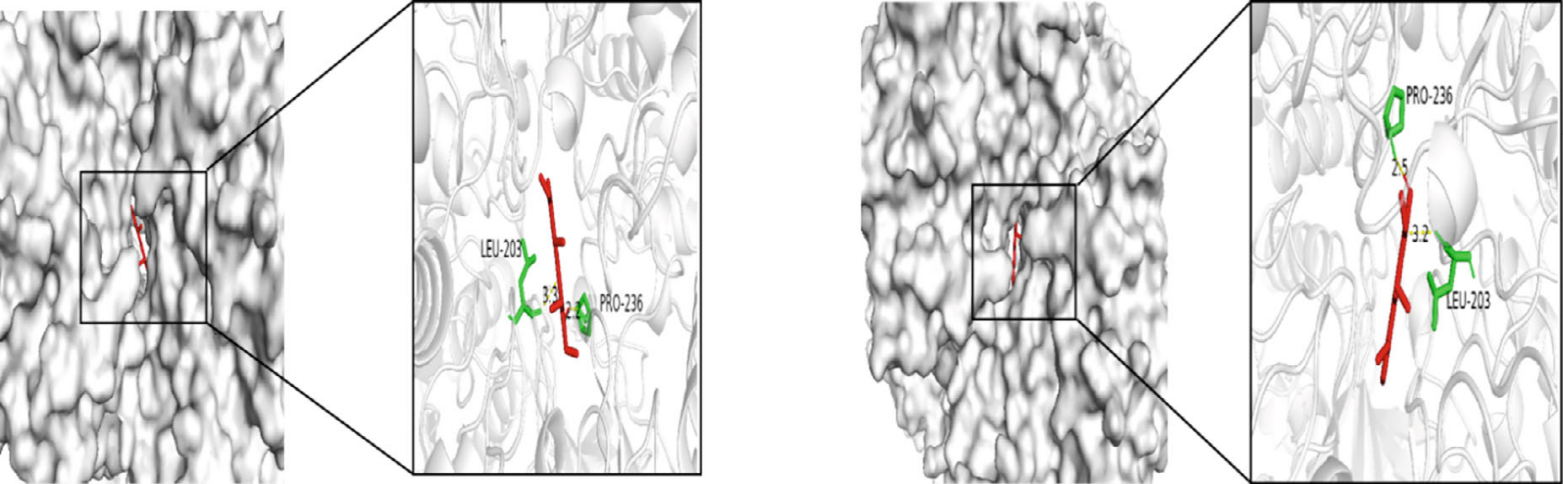
(d)

**Figure:**
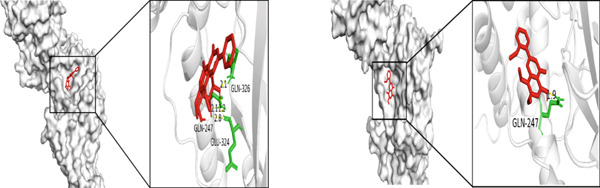
(e)

## 4. Discussion

*A. paniculata* was first recorded in the *Lingnan Caiyao* Lu alongside 480 other traditional Chinese medicinal herbs. Owing to the diverse pharmacological effects of its active components, it is widely recognized as a “natural antibiotic” and is regarded as a safe, nontoxic, and naturally “cold” traditional Chinese medicine [7]. In recent years, numerous studies have identified key active constituents of *A. paniculata* that contribute significantly to its therapeutic efficacy. For example, Kan Jang, a standardized dried extract of *A. paniculata*, has been developed for the treatment of upper respiratory tract infections and the common cold. During the 1919 influenza pandemic in India, tinctures of *A. paniculata* were reported to have played a significant role in managing the outbreak [23]. Xiyanping injection, a formulation whose main active compound is sulfonated andrographolide, is widely used in China for treating conditions such as bronchitis, tonsillitis, and bacillary dysentery [24]. In the context of influenza treatment, andrographolide derivatives have demonstrated potent antiviral activity. Notably, 14‐*α*‐lipoyl andrographolide (AL‐1) exhibits high efficacy against influenza A viruses, including H5N1 avian influenza virus [25]. Studies using both aqueous and ethanol extracts of *A. paniculata* have also shown significant antiviral properties against H5N1 [26]. Moreover, 14‐deoxy‐11,12‐dehydroandrographolide (DAP) has been reported to inhibit H5N1 replication by blocking nuclear export of the vRNP complex [27]. While most research has focused on andrographolide itself, the findings of this study suggest that flavonoid components such as Mono‐O‐methylwightin and Andrographin also possess strong anti‐influenza potential, especially through high‐affinity interactions with the core target GAPDH.

Based on network pharmacology analysis, this study identified 13 key targets through the PPI network, including TNF, IL6, AKT1, GAPDH, STAT3, JUN, ALB, and NFKB1. Among them, TNF exhibited the highest centrality (Degree = 272), highlighting its potential role as a major regulatory target. TNF is an inflammatory mediator with normal physiological functions in maintaining homeostasis and health, and also plays a role in antimicrobial immunity. It activates the NF‐*κ*B signaling pathway, promotes the aggregation and activation of inflammatory cells, and aggravates the inflammation of atherosclerosis [28, 29]. IL6 is a cytokine with multiple activities, which is involved in liver regeneration and metabolic control of the body, and can regulate the immune system and nervous system [30]. AKT is a serine/threonine protein kinase, with AKT1 being one of its three subtypes. The PI3K‐AKT signaling pathway is a core regulatory pathway in cells, governing growth, proliferation, motility, and metabolism. AKT1, a key downstream kinase in this pathway, regulates apoptosis, cell proliferation, and inflammatory responses in smooth muscle cells, and plays a role in diseases such as atherosclerosis and pulmonary fibrosis [31]. GAPDH is one of the most prominent housekeeping proteins, involved in glycolysis and various tumor‐related biological processes [32]. STAT3, a multifunctional signaling transcriptional activator, serves as a convergence point for numerous oncogenic pathways. It is extensively overactivated in both cancerous and noncancerous cells, playing a pivotal role in suppressing the expression of key immune activation regulators and promoting the production of immunosuppressive factors [33]. JUN is a basic leucine zipper (bZIP) protein, a cancer protein that regulates human tumor transformation and development, and is expressed in both immune and inflammatory cells [34]. Albumin (ALB) is associated with systemic inflammatory responses and nutritional status. It plays a role in scavenging free radicals, maintaining colloid osmotic pressure, and protecting neurons. The level of ALB correlates with the severity of renal cancer [35]. The NFKB1 gene encodes a subunit of the NF‐*κ*B (nuclear factor *κ*B) family. As a cornerstone of cellular biology, the NF‐*κ*B pathway regulates critical processes including cell proliferation, apoptosis, and stress responses. Within this pathway, NFKB1 plays a central role in tumor development [36]. As shown in Figure 3, GO functional enrichment analysis revealed that these targets are predominantly involved in inflammatory BPs. At the cellular level, the primary site of action appears to be the cytosol, while MFs are closely associated with protein binding and ATP binding activities. KEGG pathway enrichment analysis further demonstrated that the therapeutic action of *A. paniculata* may involve modulation of lipid metabolism, atherosclerosis, and oncogenic pathways. The results of molecular docking confirmed that the major flavonoid compounds exhibit strong binding affinities to the core targets, supporting their relevance in the anti‐influenza mechanism of *A. paniculata*. However, these in silico findings require further experimental validation through in vitro and in vivo studies.

## 5. Conclusion

In conclusion, this study elucidates the anti‐influenza mechanisms of *A. paniculata* through a network pharmacology and molecular docking approach. *A. paniculata* may exert its therapeutic effects against influenza by modulating core targets such as TNF, IL‐6, AKT1, GAPDH, and STAT3. These results provide a scientific basis for the application of traditional Chinese medicine in influenza therapy.

## Conflicts of Interest

The authors declare no conflicts of interest.

## Author Contributions

Lang‐ling Tian designed and performed the experiment and wrote papers; Mang‐ling Zhang helped collect the data; Cong Wang supervised this subject.

## Funding

This work was supported by research funding from the National Natural Science Foundation of China (No.82360727) and Yunnan Provincial Local Universities Joint Research Special Fund (No.202101BA070001‐114).

## Data Availability

The processed data can be obtained from the corresponding author upon request.
